# Dr. Barry, on Cow-Pock

**Published:** 1800-11

**Authors:** John W. Barry

**Affiliations:** Cork


### To Dr. PEARSON.

Dear Sir,

I Should have anfwered your polite letter (inclofing the Vaccine matter) fooner, but waited in the hope of collecting farther information with refpect to the traditional and popular knowledge of the Cow-pock. My refearches have been hitherto confined to a fmall portion of the weftern part of this country, where I have met with thirteen inftances of people who had gone through the Cow-pock, and were afterwards exempt from the Small-pox. With regard to the fuppofed origin of the difeafe, it was never fufpected in this country *to be derived from the grease of the horse's heel*: Indeed, the bare proof of the exiftence of the Cow-pock in this country, feems to me a fufficient refutation of the only hypothetical part of an inveftigation conducted with uncommon ingenuity, and completely fatisfactory in the principal objects for which it was undertaken, viz. the fuperior mildnefs of the Cow-pock, and its power of deftroying the fufceptibility of the conftitution to the Small-pox. Women alone are employed in milking cows in this country; in this office men never interfere, as they would think themfelves difgraced by engaging in any rural occupation, in which the other fex are ufually employed.

I now proceed to relate fome of the cafes, deferring any further remarks until a few of thefe are detailed.

Cornelius Creedon, æt. 60, (a labourer in the employment of Samuel Townfhend, Efq. Firmount) feveral years ago flept with a cow-boy, who took the Shinach, (Cow-pock) from handling the teats of cows affected with it. He had fhivering, general uneafinefs, and ficknefs at the ftomach, which fubfided on the appearance of an eruption in his neck, breaft, and arms; the auxillary glands were fwelled and very painful. In ten years after he was inoculated with the Small-pox, and has fince, during three fucceffive inoculations of this difeafe in his family, handled and nurfed his children without the leaft effect. He, as well as his entire neighbourhood, attributed his efcape to his previoufly having had the Cow-pock. The cow-boy, as far as he recollects, got the difeafe from rubbing the fore teats of the cows with fome application, which he believes to have been burnt butter: is fure it was from the cow-boy he was infected, as he did not himfelf handle the cows. His fifter had the diforder at the fame time, and has been fince alfo inoculated, without taking the fmall-pox.

Ellen Goggin, (wife to a tenant of Mr. Townfhend) feventeen teen years ago, after previous ficknefs at ftomach, and fymptoms of general fever, had a copious eruption of Cow-pock on her hands. The cows were much afflicted with it at the time, and their milk was in confequence much leffened. The ficknefs fhe defcribes as being more fevere than any fhe had ever experienced; but fhe was greatly relieved by the eruption. By frequent handling, fhe communicated the difeafe to an infant whom fhe was fuckling; but the eruption, though copious, did not fuppurate, and fhe could not obferve any figns of general difturbance in him. In two years after, this boy and a younger brother were inoculated for the Small-pox; they lay together during the progrefs of the difeafe; his brother fhewed a great quantity of Small-pock, but he had no fign of it either at the arm or elfewhere. Three more of the family were lately inoculated, with one of whom he flept, though he had the Small-pox, which, however, he did not take. During thefe inoculations, the mother conftantly attended the children, and handled them daily, without taking the Small-pox. She attributes her own efcape, as well as that of her fon, to their having had the Cow-pock, as above related, and has often heard and feen inftances of fimilar exemption produced by this difeafe.

Can it be inferred from thefe caufes, that the difeafe is infectious in its natural ftate? In the inftance of Goggin, it is eafy to conceive, that the woman might have handled the child with her hands covered with fluid matter from the cows' teats. May not the fuperior violence of the difeafe render it communicable by contact in its natural ftate? In the inoculated Cow-pock I can determine this point, if it were not fufficiently proved already. I inoculated fifty children, not above twenty of whom fhewed the marks of infection at the arm at the ufual period. The infected children continued to fleep with the uninfected as ufual; yet, not one of the latter took the diforder, which I was forced to communicate to them by a fecond inoculation.

Johanna Sullivan, æt. 50, (cook-maid at Dr. Richard Walfh's of this city) when fhe was 13 years of age, was brought with a number of other children to a dairy, for the purpofe of being infected with a diforder of cows called the Shinach, which, by the general belief of the neighbours, would fecure for ever fuch as took it from the Small-pox. She and the other children were made to fqueeze the cows' teats till their hands and fingers were covered with the fluid matter of the diforder. She recollects that a number of the children did not take the difeafe; fhe, however, had one pimple on her hand, which inflamed and fuppurated. When fhe was twenty years of age fhe

ſhe was twice inoculated by Mr. Godwin, an apothecary at Bantry, without effect; but on hearing from her mother that ſhe had the diſeaſe above-mentioned, he declined inoculating her a third time, alleging that there was not the ſmalleſt danger of ever taking the Small-pox, as he could aver from experience. She has ſince reſided in Cork, where ſhe was frequently expoſed to Small-pox, particularly about eleven years ago, when the grand-children of the late Mr. Attiwell Hayes, with whom ſhe then reſided, were inoculated. In order, as ſhe ſaid, to *be ſure of herſelf*, ſhe lay with the children four nights in the height of the eruption, but did not take the diſeaſe. As my letter is already of unconſcionable length, I ſhall conclude my account of the natural Cow-pock with the following extract of a letter from a lady of reſpectable connections; ſome of whom had experienced the Cow-pock in early life. This communication is worthy of attention, no leſs from the nature of the information, than from the authority from whence it is derived.

—— "It is thirty years ſince my mother had the Cow-pock. She recollects having had two pimples on one hand, which were much inflamed, and afterwards went on to ſuppuration. She has been inoculated frequently ſince, and expoſed to the infection of Small-pox in various ways, without taking it, which has been attributed to her having had the Cow-pock, univerſally known among our farmers by the name of Shinach. I was laſt night ſpeaking to my grand-mother on the ſubject. She had the Cow-pock fifty years ago. Her account agrees with my mother's. She had the eruption on one hand, and, ſhe ſays, ſhe never in her life experienced ſo much pain as while the inflammation laſted. She has never been inoculated, but was very often expoſed to the Small-pox without taking it. At the time ſhe had the diſeaſe, there was ſcarcely a Spring that the cows were not affected with it; and it was ſo univerſally believed that thoſe who took it were ever after exempted from the Small-pox, that people expoſed themſelves as much as poſſible to it. My grand-mother, who is above eighty years old, ſays, that the ſame opinion always prevailed in this country."

Other accounts agree, that the diſtemper is not near ſo frequent as it was forty or fifty years ago. It appeared, however, in this neighbourhood laſt month, but declined before I had an opportunity of ſeeing it. In one part of the country, where there are no traces of it except in the memories of ſome very old people, they account for its diſappearance in the following manner: They aſſert, that it was firſt produced by the bite of a certain inſect, the breed of which has been now de-

ſtroyed ftroyed by the *frogs*, which are known to be of foreign origin, and, on this account, retain their Englifh name. I mention this, to fhew that the Irifh are not wanting in conjecture on the origin of the diftemper as well as the Englifh. This opinion of the difeafe, however, is not general.

The Irifh name of the difeafe (Shinach) belongs to the old written language of the country, and has no relation to the modern colloquial dialect; this I confider a ftrong proof of the antiquity of the diftemper. It is evident from the facts I have ftated, and from the remainder which I have myfelf collected, or received from friends, that the belief of the anti-variolous power of the difeafe is as ancient as that of the exiftence of the difeafe itfelf. That this power is not *fugitive* but *permanent*, all thefe cafes fully prove; and thus an argument againft the new inoculation, which has been ufed in this city as well as in England, receives a fatisfactory anfwer.

In all the cafes I have met with, the fever preceded the eruption, and was relieved by it.

I received fome vaccine infection from Dr. Bradley, previous to my having received your letter, as I have acknowledged in a communication to the Phyfical Journal. With the thread received from him, I was fuccefsful in procuring an abundance of infection. I gave your thread to my friend Dr. Sugrue, who had equal fuccefs. He has extended the inoculation to 50 children. The number I have inoculated amounts to 250, not above three of whom had eruptions, which difappeared without fuppurating. I have not inoculated above twelve with the Small-pox, the lower order having fome fuperftitious notions, which prevent them from agreeing to a fecond inoculation. I felected five of thefe, from their not having exhibited the leaft appearance of conftitutional fever, though the progreffive ftate of the puncture at the arm had been exactly fimilar to the other cafes. Two of the five were adults, and therefore capable of defcribing any fymptoms they might have experienced. The matter I ufed was frefh taken from a child covered with variolous eruption in a ftate of fuppuration. The punctures, after looking inflamed for three or four days, withered without forming a puftule. Hence I would infer, that it is the ftate of the arm alone we fhould regard, particularly as fever is produced in children often from the flighteft caufes. I have the pleafure to acquaint you that moft of the Phyficians of refpectability in this city are fully convinced of the efficacy of the Cow-pock. I have fupplied many of them with infection; among others, Dr. Longfield, who has inoculated his only grand-child, the infant fon of Mr. Leflie, the banker. From Dr. Longfield's age, the refpectable rank he holds in his profeffion, profeſſion, and his love of ſcience, his example muſt have great weight with the public. To remove ſome objections which have been advanced againſt the inoculation, and, if poſſible, to extend its benefits as widely as poſſible, I have it in contemplation to addreſs the public in a ſmall pamphlet in a popular ſtyle. This ſeems neceſſary, as very few of the original works on the ſubject have yet reached this country, and therefore objections, however weak, muſt have their effect when they remain unanſwered. By this means I ſhall alſo, I truſt, be enabled to collect more numerous facts on the natural Cow-pock. If you have any new information on the ſubject, I ſhould be obliged by the communication of it. If you have no particular uſe for the information I tranſmit you, I ſhall thank you to ſend it to Dr. Bradley for publication, if he thinks proper. With him I beg you will accept of my acknowledgments for the infection, which promiſes incalculable benefits to this part of Ireland. I remain, dear Sir,

Your ſincere humble ſervant,

*Cork, Oct.* 16, 1800. JOHN W. BARRY.

P. S. Dr. O'Leary, your late pupil, is arrived, and informed me of your wiſh to hear from me, which induced me to write ſooner than I intended.

